# Methoxistasis: Integrating the Roles of Homocysteine and Folic Acid in Cardiovascular Pathobiology

**DOI:** 10.3390/nu5083235

**Published:** 2013-08-15

**Authors:** Jacob Joseph, Joseph Loscalzo

**Affiliations:** 1 Department of Medicine, VA Boston Healthcare System, Boston, MA 02132, USA; 2 Department of Medicine, Brigham and Women’s Hospital, Boston, MA 02115, USA; E-Mail: jloscalzo@partners.org

**Keywords:** methionine, homocysteine, folic acid, cardiovascular disease, heart failure, nutrition, pyridoxine, cobalamin, nutrition, redox, methylation

## Abstract

Over the last four decades, abnormalities in the methionine-homocysteine cycle and associated folate metabolism have garnered great interest due to the reported link between hyperhomocysteinemia and human pathology, especially atherothrombotic cardiovascular disease. However, clinical trials of B-vitamin supplementation including high doses of folic acid have not demonstrated any benefit in preventing or treating cardiovascular disease. In addition to the fact that these clinical trials may have been shorter in duration than appropriate for modulating chronic disease states, it is likely that reduction of the blood homocysteine level may be an oversimplified approach to a complex biologic perturbation. The methionine-homocysteine cycle and folate metabolism regulate redox and methylation reactions and are, in turn, regulated by redox and methylation status. Under normal conditions, a normal redox-methylation balance, or “methoxistasis”, exists, coordinated by the methionine-homocysteine cycle. An abnormal homocysteine level seen in pathologic states may reflect a disturbance of methoxistasis. We propose that future research should be targeted at estimating the deviation from methoxistasis and how best to restore it. This approach could lead to significant advances in preventing and treating cardiovascular diseases, including heart failure.

## 1. Introduction

Since the seminal report by Kilmer McCully based on autopsy analysis of two subjects with homocystinuria and very high plasma homocysteine levels who died from vascular complications [1], a significant body of research has investigated the link between hyperhomocysteinemia and cardiovascular disease. While observational studies have demonstrated a link between an elevated plasma level of total homocysteine and cardiovascular disease, clinical trials utilizing low and high doses of folic acid and vitamin B_12_, which are involved in remethylation of homocysteine to methionine by methionine synthase, and vitamin B_6_, which acts as a cofactor in the transsulfuration of homocysteine to cystathionine and cysteine, have not shown any clinical benefit [2]. Most of these studies did not select patients based on a significantly elevated plasma homocysteine level, an approach which may have influenced the results. Since folate fortification programs in North America have been circumstantially associated with a reduction in stroke rates [3], it is possible that the clinical trials were too short in duration to reverse established or progressive cardiovascular pathology. However, since some studies have shown deleterious effects of B-vitamin supplementation [4,5,6,7,8], it is also possible that the current approach of lowering plasma homocysteine to reduce cardiovascular risk is inadequate. Current evidence suggests that an abnormal plasma homocysteine level “uncovers” an underlying perturbation of the methionine-homocysteine cycle and folate metabolism, but more detailed analysis is needed to delineate the underlying pathobiology [9].

The methionine-homocysteine cycle regulates both methylation reactions and redox balance. Methylation reactions are dependent on the levels of the methyl donor *S*-adenosyl methionine (SAM) and methylation inhibitor *S*-adenosyl homocysteine (SAH), both of which are generated by reactions of the methionine-homocysteine cycle [10]. The transsulfuration pathway of homocysteine converts methionine to cysteine, which, along with its derivative glutathione (γ-glutamyl cysteinyl glycine), are the major extra- and intra-cellular antioxidants, respectively. Nutritional intake of folate and of the sulfur-containing amino acids methionine and cysteine also affects various enzymatic reactions of the methionine-homocysteine cycle and associated one-carbon metabolism, and, thereby, redox-methylation balance [11,12]. Among cardiovascular diseases, heart failure is likely to be more influenced by changes in redox methylation balance since deficient nutritional intake, catabolic state, and loss of micronutrients due to therapy are all prevalent in patients with heart failure [13].

## 2. Methionine-Homocysteine Cycle Regulates Redox and Methylation Reactions

### 2.1. Overview of the Methionine-Homocysteine Cycle and Sulfur-Containing Amino Acid Metabolism

**Figure 1:**
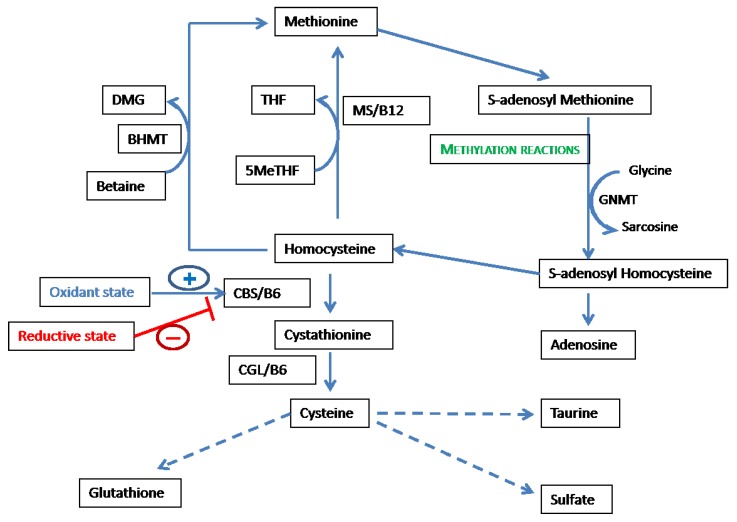
Methionine-homocysteine cycle is closely linked to redox balance, methylation, and sulfur amino-acid metabolism. Abbreviations: THF—tetrahydrofolate; 5MeTHF—5′ methyl tetrahydrofolate; BHMT—betaine homocysteine methyltransferase; DMG—dimethyl glycine; MS—methionine synthase; GNMT—glycine *N*-methyl transferase; CGL—cystathionine γ-lyase; and CBS—cystathionine β synthase.

The sulfur-containing amino acids are crucial to health (reviewed in detail in [11]). Dietary methionine is an essential amino acid, and renders cysteine semi-essential since the sulfur atom of methionine can be utilized to synthesize cysteine via homocysteine, which enters the transsulfuration pathway from the methionine-homocysteine cycle. As shown in Figure 1, the cycle is initiated by the conversion of methionine to the universal methyl donor SAM. This reaction utilizes ATP and is catalyzed by the enzyme methionine adenosyl transferase (MAT). SAM donates the methyl group utilized in all methylation reactions except remethylation of homocysteine [14]. Various molecules, including DNA, RNA, histones and other proteins, as well as guanidinoacetate, are methylated utilizing methyl groups derived from SAM. The methylation of glycine to sarcosine, catalyzed by glycine *N*-methyl transferase (GNMT), modulates SAM levels and, thereby, methyl group supply [15]. *S*-adenosyl homocysteine, generated by removal of the methyl group, is hydrolyzed by *S*-adenosyl homocysteine hydrolase (SAHH), which catalyses the hydrolysis of SAH to produce adenosine and homocysteine [16,17]. Homocysteine can undergo remethylation or transsulfuration, and the distribution between these two processes is crucial to the regulation of redox and methylation reactions. Remethylation can occur by the acceptance of methyl groups via two reactions. Methionine synthase (MS), which utilizes cobalamin/vitamin B_12_ as a cofactor catalyzes the donation of a methyl group from 5′-methyl tetrahydrofolate (5meTHF) to homocysteine to form methionine. An alternate pathway of remethylation is catalyzed by betaine homocysteine methyl transferase (BHMT), which utilizes betaine as the methyl donor [18]. Transsulfuration is initiated by the irreversible conversion of homocysteine and serine to cystathionine by the enzyme cystathionine β synthase (CBS), which utilizes vitamin B_6_/pyridoxal phosphate (PLP) as a cofactor [19]. Cystathionine γ lyase (CGL) catalyzes the next step in transsulfuration, *i.e.*, the conversion of cystathionine to cysteine with the release of α-ketobutyrate, which enters the tricarboxylic acid cycle, and ammonia. Cysteine is a substrate for the rate-limiting step in glutathione (γ-glutamyl cysteinyl glycine) synthesis, *i.e.*, the formation of γ-glutamyl cysteine, a reaction that is catalyzed by glutamate cysteine ligase [20]. In addition to being utilized for synthesis of protein and glutathione, cysteine undergoes catabolic conversion to taurine and sulfate, which are also essential for normal health [21]. The total intake of methionine and cysteine should normally equal elimination—predominantly as sulfate and taurine—to maintain sulfur balance. Hence, the methionine-homocysteine cycle and associated pathways are crucial to, and influenced by, various biological compartments, such as methylation, redox balance, nucleic acid synthesis, and also sulfur balance.

### 2.2. Methionine-Homocysteine Cycle and Redox Potential

Both intracellular and extracellular redox status are crucial to homeostasis (reviewed in detail in [22,23]). The major redox buffers are glutathione, cysteine, and thioredoxin; the thiol groups in these molecules are readily oxidized and, hence, can buffer changes in redox state. Several cellular biologic processes, such as proliferation, apoptosis, and macromolecular synthetic functions, are regulated by redox signaling. Glutathione is the major intracellular redox buffer, present in millimolar concentrations, with a cytosolic ratio of reduced-to-oxidized glutathione disulfide (GSH/GSSG) of 50:1 [22]. The redox state varies between cellular compartments; e.g., the endoplasmic reticulum has a GSH/GSSG ratio of 2:1, suggesting a higher oxidant state required for protein folding. Thioredoxin and cysteine are the other intracellular thiol redox buffers, present only in micromolar concentrations. Extracellular redox potential is also crucial to homeostasis: the functions of extracellular and membrane associated proteins, including receptor proteins, are regulated by redox-mediated structural changes. Cysteine is the major extracellular redox buffer and is present more in the oxidized form, with a ratio of reduced cysteine to oxidized cysteine disulfide in plasma of approximately 1:4.

Cysteine is derived from the transsulfuration pathway, from the diet, or from protein catabolism (in the fasting state). The rate-limiting enzyme for transsulfuration, CBS, is not present in most tissues except the liver and kidney; in fact, transsulfuration is non-existent in cardiovascular tissues [24].

Hence, most tissues, including cardiovascular tissues, are dependent on transport and cellular uptake of cysteine for synthetic functions and for glutathione synthesis. Cysteine synthesized via transsulfuration can be delivered to other tissues as reduced cysteine (cysteine disulfide cannot be taken up by most cells) or is derived from the catabolism of glutathione. In fact, most of the transport of cysteine between tissues occurs in the form of glutathione, which is hydrolyzed by plasma and extracellular enzymes to cysteine for cellular uptake [25]. Hence, the methionine-homocysteine cycle is the major system that regulates the concentration of the major intra- and extracellular redox buffers. Therefore, the methionine-homocysteine cycle itself can act as a buffer to regulate redox status in the face of varying dietary intake of aminoacids and oxidant stress.

Abnormalities in the methionine-homocysteine cycle, whether due to genetic, nutritional, or other factors, can elevate the plasma level of homocysteine. Studies from our laboratories and those of others over the last four decades have shown an association of elevated plasma homocysteine levels with oxidant stress-induced pathology in the cardiovascular system [26,27,28]. Several mechanisms have been postulated for the increased oxidant stress observed in these hyperhomocysteinemic states: direct auto-oxidation of homocysteine generating reactive oxygen and nitrogen species; induction of pro-oxidant enzymes such as NADPH oxidase; and suppression of antioxidant enzymes such as glutathione peroxidases [29]. Preclinical studies have also shown that increasing antioxidant capacity by genetic or nutritional methods can attenuate hyperhomocysteinemia-induced vascular and myocardial pathology [28,30]. What is not clear is whether oxidant stress is the sole pathogenic mechanism in hyperhomocysteinemia-induced pathology in humans.

### 2.3. Feedback of Redox Status on the Methionine-Homocysteine Cycle

As may be expected based on the close relationship of the methionine-homocysteine cycle to redox state, all of the major enzymes of the cycle are regulated by redox status. Activity of the enzyme CBS is a major node in the methionine-homocysteine cycle that regulates the partitioning of homocysteine generated from methylation reactions into the transsulfuration pathway or remethylation back to methionine. CBS has an *N*-terminal segment that contains a regulatory heme binding domain [31,32]. The oxidation state of the heme moiety is sensed by the phosphorus nucleus of the enzyme cofactor pyridoxal phosphate, which is bound to the catalytic domain [33]. Oxidation of the heme moiety increases CBS activity, while reduction decreases transsulfuration [34,35]. Methionine synthase, the ubiquitous homocysteine remethylating enzyme, is also redox-sensitive. The enzyme cofactor cobalamin/vitamin B_12_ is highly reactive and is vulnerable to inactivation by oxidation. This oxidative lability of MS is counterbalanced by reductive reactivation catalyzed by flavoprotein oxidoreductases, such as methionine synthase reductase [36]. The alternate homocysteine remethylating enzyme betaine homocysteine methyl transferase is also a labile enzyme that requires thiol mediated reduction to maintain catalytic activity [37]. Mosharov and colleagues have demonstrated the crucial role of redox state on the partitioning of the “key junction metabolite” homocysteine into the transsulfuration and remethylation pathways. In human liver cells, they showed that the addition of the oxidants hydrogen peroxide and tertiary-butyl hydroperoxide linearly increased the conversion of homocysteine to cystathionine [34]. Other enzymes of the pathway are also regulated by redox balance; e.g., MAT activity is reduced under conditions of oxidant stress [38]. Generally, the transsulfuration pathway appears to be activated by an increase in oxidant molecules, with the reverse occurring in the case of enzymes involved in remethylation of homocysteine and other methylation reactions.

### 2.4. Methionine-Homocysteine Cycle and Methylation Reactions

*S*-adenosyl methionine is the methyl donor for all methylation reactions in the body except remethylation of homocysteine. Thus, methylation reactions are dependent on the synthesis of SAM, a reaction catalyzed by MAT and requiring methionine and adenosine triphosphate (ATP) as substrates. MAT is present in three isoforms [39]. MAT2 is present in all tissues, has a high affinity for methionine, and is inhibited by SAM; hence, most tissues have a maximal level of SAM synthesis [40]. The liver has two isoforms of MAT: MAT1, which has a higher *K*_m_ than MAT2 and is less inhibited by SAM, and MAT3, which has a low affinity for SAM and is activated by SAM. Hence, the liver, via MAT3, allows the body to respond to high methionine or SAM levels. Thus, MAT, via synthesis of and feedback by SAM, is a major regulator of methyl balance and methylation reactions.

A large number of methylation reactions occur during normal metabolism resulting in the transfer of methyl groups to sulfur, nitrogen, or oxygen atoms in a diverse array of biological compounds. Approximately 13–14 mEq/day of methyl groups are utilized in methylation reaction, with the bulk (10 mEq/day) being used in the conversion of guanidinoacetate to creatine [14,41]. Major methyl acceptors regulated by methylation include DNA, RNA, cathecholamines, phospholipids, and proteins. Methyltransferases are specific for each methyl acceptor, and generally have high affinity for SAM [42,43]. Glycine-*N*-methyl transferase (GNMT) catalyses the conversion of glycine to *N*-methyl glycine (sarcosine), has a high *K*_m_ (low affinity) for SAM, and is only weakly inhibited by SAH [44]. Hence, the cycling between glycine and sarcosine is utilized by the body to optimize methyl group supply and methylation reactions.

As described above, the local concentration of SAM is crucial for methylation reactions; however, methyltransferases, including DNA methyltransferases, have low *K*_m_ for SAM. SAH, however, is a potent inhibitor of all methyltransferases [42,43]. Hence, the ratio SAM/SAH is considered a major regulator of methylation reactions. SAM/SAH ratio or “methylation potential” is generally less than inhibitory for methyltransferases under normal conditions. Homocysteine accumulation significantly affects SAH levels and, hence, methylation potential. The equilibrium of the hydrolysis reaction which converts SAH to homocysteine and adenosine, catalyzed by SAH hydrolase (SAHH), favors the formation of SAH. Hence, rapid removal of homocysteine is necessary for the hydrolysis of SAH [16]. Therefore, if conversion of homocysteine by remethylation and transsulfuration or by its transport is impaired, methylation potential will be altered and methylation reactions inhibited. The importance of SAH and the SAM/SAH ratio to methylation has been proven by clinical studies. For example, Yi and colleagues have demonstrated that plasma SAH levels were associated with lymphocyte global DNA hypomethylation in healthy, young women [45].

## 3. Folate, Methionine-Homocysteine Cycle, and Redox-Methylation Balance

### 3.1. Overview of Folate-Dependent One Carbon Metabolism

**Figure 2:**
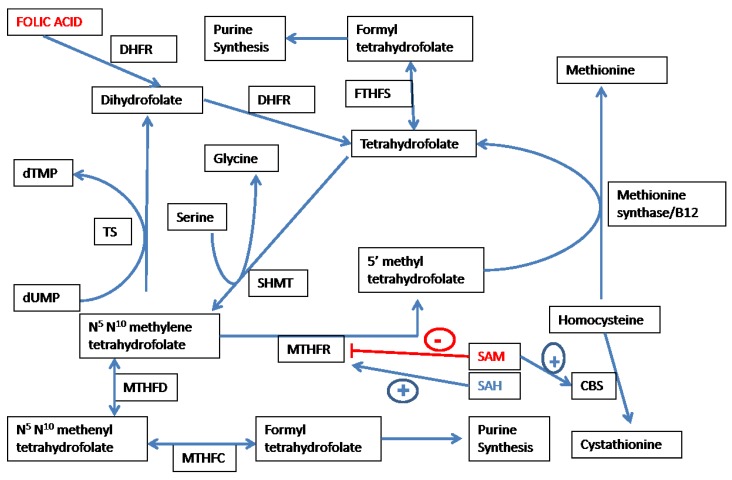
Folate metabolism and methionine-homocysteine cycle. Abbreviations: DHFR—dighydrofolate reducatse; SHMT—serine hydroxymethyl transferase; MTHFR—methylene tetrahydrofolate reductase; MTHFD—*N*^5^,*N*^10^-methylene tetrahydrofolate dehydrogenase; MTHFC—*N*^5^,*N*^10^-methenyl tetrahydrofolate cyclohydrolase; FTHFS—Formyl tetrahydrofolate synthase; CBS—cystathionine beta synthase; TS—thymidylate synthase; UTP—deoxy uridine monophosphate; dTMP—deoxy thymidine monophosphate; SAM—*S*-adenosylmethionine; and SAH—*S*-adenosyl homocysteine.

Various forms of folate are utilized in the transfer of one carbon moieties, such as methyl (–CH_3_), methylene (–CH_2_–) and formyl (–CHO–) groups in metabolic pathways [12]. As shown in Figure 2, *N*^5^,*N*^10^-methylene tetrahydrofolate (MTHF) is irreversibly converted by the FAD-dependent enzyme methylene tetrahydrofolate reductase (MTHFR) to the methyl donor 5-methyl-tetrahydrofolate (5MeTHF). 5MeTHF provides the methyl group for the MS/cobalamin dependent remethylation of homocysteine to methionine. Tetrahydrofolate (THF) so generated can be converted to its immediate precursor (MTHF) by the enzyme serine hydroxyl methyl transferase (SHMT) with the concomitant conversion of serine to glycine. MTHF donates the methyl group involved in the methylation of deoxy-uridine monophosphate to deoxythymidine monophosphate (catalyzed by thymidylate synthase). Dihydrofolate produced during thymidylate synthesis is reduced by dihydrofolate reductase to THF. Another important function of folate involves transfer of the formyl group during the synthesis of purines. *N*^5^,*N*^10^-methylene tetrahydrofolate dehydrogenase (MTHFD) catalyzes the conversion of MTHF to formyl THF and the transfer of the carbon atom as the formyl group during the synthesis of purines. Hence, folate metabolism via transfer of various one-carbon moieties is linked both to DNA synthesis and DNA methylation.

### 3.2. Folate Affects Redox-Methylation Balance via the Methionine-Homocysteine Cycle

Figure 1, Figure 2 demonstrate the key role of folate metabolism in maintaining the balance between redox and methylation status in the body. The methyl group of 5MeTHF is synthesized *de novo* from the carbon atom of serine as shown in Figure 2. *N*^5^,*N*^10^-methylene tetrahydrofolate is irreversibly converted to 5MeTHF by the key enzyme MTHFR. As shown in Figure 2, SAM and SAH effect negative and positive feedback, respectively, on MTHFR [46,47]. 5MeTHF is a negative regulator of GNMT, and a reduction in 5MeTHF levels consequent to SAM-induced suppression of MTHFR activity, activating GNMT and leading to the utilization of excess methyl groups in the methylation of glycine to sarcosine [48,49]. As mentioned above, the key “junctional” enzyme CBS has an *N*-terminal regulatory heme binding domain that responds to changes in redox state. The *C*-terminal domain exerts an inhibitory effect on the catalytic domain under normal conditions. SAM binds to the *C*-terminal domain of CBS and abolishes its inhibitory effect on the catalytic site, thereby acting as an allosteric activator of CBS [50]. Hence, as shown in Figure 1, Figure 2, folate interacts with key intermediates of the methionine-homocysteine cycle to regulate the methyl supply by directing the metabolic fate of homocysteine to remethylation or transsulfuration.

## 4. Clinical Evidence Connecting Methionine-Homocysteine Cycle, Folate Metabolism, and Methylation

Variable results have been obtained in studies of the effect of folate supplementation on DNA methylation. Two studies took into account a polymorphism in the gene for MTHFR, *i.e.*, MTHFR C677T, which results in MTHFR CC, CT and TT genotypes, with a significant (≥50%) reduction in the activity of MTHFR and a commensurate increase in plasma homocysteine levels observed in MTHFR TT compared to MTHFR CC genotype [51,52]. Jung and colleagues studied the effect of folic acid (0.8 mg) administered for three years on DNA methylation stratified by MTHFR genotype [53]. There was no effect of folate supplementation on global DNA methylation, and MTHFR genotype did not affect treatment effect. Another similar study evaluated the effect of high-dose folic acid (5 mg/day) in patients with the MTHFR TT polymorphism [54]. In this study, high-dose folate reduced plasma homocysteine levels by almost 50% and increased the SAM/SAH ratio; however, there was no effect on global DNA methylation. Hence, supplementing folate even in selected patients with hyperhomocysteinemia due to a folate metabolic defect does not seem to alter DNA methylation, despite favorable effects on methylation potential.

A larger three year study of the utility of aspirin and folic acid for prevention of colorectal adenomas evaluated global methylation in colonic biopsies by estimating the level of methylation of the long interspersed nuclear element (LINE-1) [55]. In this study, there was no effect of folate supplementation on global DNA methylation. In contrast, a similar study but of shorter duration (10 weeks of folic acid 0.4 mg/day) demonstrated that folate treatment resulted in an increase in global DNA methylation in leucocytes and colonic mucosa, as well as a decrease in the plasma homocysteine level [56]. Rampersaud and colleagues studied the effect of folate depletion and repletion in healthy post-menopausal women [57]. Folate depletion led to hypomethylation of DNA; however, seven weeks of folate repletion did not normalize DNA methylation levels. These data suggest that the effects of folate may be influenced by duration of treatment.

Overall, these data suggest that the effect of folate is not predictable; effects on DNA methylation are complex and influenced by duration of treatment, dose of folate, tissue involved, and baseline demographic features, such as age. Furthermore, effects on specific genes may be different from that observed on global DNA methylation. Another variable is the presence of a non-folate dependent pathway that effects the remethylation of homocysteine, *i.e.*, the alternate remethylation pathway, which utilizes betaine as the methyl donor and results in the production of dimethylglycine. A recent report demonstrated that the plasma dimethylglycine level was independently related to the risk of myocardial infarction [58]. Hence, a major issue that needs to be addressed is how the two pathways of remethylation and the transsulfuration are coordinately regulated in health, and how this regulation might be altered in disease states.

## 5. Clinical Evidence Connecting the Methionine-Homocysteine Cycle, Folate Metabolism and Redox Balance

Wotherspoon and colleagues studied the response of insulin-dependent diabetics to folate treatment [59]. Although plasma homocysteine level was reduced by 25% in folate-treated subjects compared to the placebo group, no significant changes were observed in markers of oxidant stress. A study by Alvares-Delfino and coworkers examined the effects of folate in patients with end-stage kidney disease on dialysis [60]. The authors reported that folic acid treatment normalized plasma homocysteine levels and increased plasma antioxidant capacity; however, hydroperoxide levels did not change. Antoniades and colleagues examined the effects of 5MeTHF delivered by intravenous infusion on saphenous veins and internal mammary arteries in patients undergoing coronary bypass surgery [61]. Studies of vascular tissue revealed that 5MeTHF reduced superoxide levels. A study by Ullegadi and colleagues in acute ischemic stroke patients compared the effect of B-vitamin therapy (folate and vitamins B_2_, B_6_, and B_12_) to standard treatment [62]. Compared to the control group, B-vitamin treatment reduced plasma malondialdehyde levels without a significant decrease in plasma homocysteine levels. A larger study was conducted by the same group adding antioxidant treatment (vitamins C and E) alone or in combination with B-vitamin treatment [63]. After two weeks of treatment, B-vitamin treatment resulted in significant reductions in plasma homocysteine and plasma malondialdehyde levels. Interestingly, there were no additional effects on oxidant levels in a group given B vitamins and anti-oxidants (vitamins E and C). Childs and colleagues examined the effect of folic acid alone on plasma glutathione levels in patients with non-insulin dependent diabetes mellitus. Oral folic acid treatment increased glutathione levels significantly while concomitantly reducing homocysteine levels. The increase in glutathione was correlated with levels of vitamin B_6_ (which was not supplemented), suggesting that increased transsulfuration was induced by folate in the presence of an adequate supply of the cofactor vitamin B_6_. Another study by Moat and colleagues examines the effect of increased folate intake via folate rich foods in healthy subjects [64]. Increasing folate intake reduced plasma homocysteine levels and induced an increase in total antioxidant capacity that was of borderline significance. Andersson and colleagues measured the B vitamins (folate, B_6_, B_12_) on homocysteine redox status (ratio of reduced-to-total homocysteine) in patients with acute coronary syndromes and elevated plasma homocysteine levels [65]. Even though B-vitamin therapy normalized plasma total homocysteine, the low ratio of reduced-to-total homocysteine observed in these patients was not normalized by B-vitamin therapy, suggesting a lack of effect on redox balance despite a reduction in total homocysteine levels. A similar study by Doshi and colleagues examines the effect of folic acid in patients with coronary artery disease. Folate improved endothelial function measured by flow-mediated dilatation, which was not correlated with the extent of homocysteine reduction. Furthermore, total antioxidant capacity or plasma markers of oxidant stress did not change with folate supplementation.

Cumulatively, current data suggest that B vitamins including folate have variable effects on redox status that are not easily captured by standard assays, and are not well correlated with homocysteine lowering.

## 6. Clinical Trials of B-Vitamin Supplementation in Cardiovascular Disease

Several studies of B-vitamin supplementation have been reported over the last two decades. A major factor that may have influenced the outcomes of these trials is the introduction of folate fortification in North America in the middle 1990s. Yang and coworkers compared stroke mortality rates before and after folate fortification in North America compared to the population of England and Wales, where folate fortification is not mandated [3]. In the U.S. population, concomitant with a rise in folate levels and a decrease in homocysteine levels, the rate of decline in stroke mortality after initiation of folate fortification was significantly greater than that observed before folate fortification. A similar trend was observed in Canada, as contrasted with a lack of any change in stroke mortality in England and Wales during the same time period. Although this study does not imply causality, these data may indicate that folate given as a primary prevention strategy may have beneficial effects on cerebrovascular disease.

Several, large, randomized trials of B-vitamin therapy as a secondary prevention strategy in subjects with established cardiovascular disease or at significant risk for cardiovascular disease have been reported in the last 10 years. Regardless of whether randomized clinical trials of homocysteine lowering utilizing B vitamins were conducted in fortified or non-fortified populations, they have been uniformly neutral in terms of their impact on preventing cardiovascular outcomes [9]. The Vitamin Intervention of Stroke Prevention trial tested the effect of high- and low-dose B-vitamin therapy in subjects who had suffered cerebral infarction [66]. Although baseline homocysteine levels were correlated with outcomes, there was no effect of homocysteine-lowering therapy on recurrent stroke. Two trials in the Norwegian population, where folate fortification of food is not mandatory, assessed the effect of vitamin B_6_ alone, folate + vitamin B_12_, and folate + B_6_ + B_12_ in subjects who had suffered an acute myocardial infarction [4] or were undergoing coronary angiography for suspected coronary artery disease or aortic stenosis [67]. Both studies did not demonstrate any benefit of B-vitamin therapy; in fact in the NORVIT study in subjects with acute myocardial infarction, an increased risk of cardiovascular endpoints was noted in the group treated with folate, B_6_, and B_12_. A combined analysis of these two Norwegian studies also did not show any benefit of B-vitamin therapy; in fact, the analysis demonstrated that combined treatment with folic acid and vitamin B_12_ treatment was associated with an increased risk of major adverse cardiac events and cardiovascular mortality [7]. In the Heart Outcomes Prevention Evaluation-2 (HOPE-2) study, older subjects with documented vascular disease or who were at risk for vascular disease were randomized to treatment with folate/B_6_/B_12_ or placebo [68]. No benefit was observed on cardiovascular outcomes over a 5-year follow-up period. In a study that enrolled women at risk of cardiovascular events, the combination of folate, B_6_, and B_12_ did not affect cardiovascular event rate [69]. Advanced chronic kidney disease is associated with elevated plasma homocysteine level and increased risk of cardiovascular disease. A trial conducted in the U.S. evaluated the effect of combination high dose B-vitamin therapy on cardiovascular outcomes in men with advanced kidney disease and plasma homocysteine level >15 μmol/L [70]. Other studies in subjects with chronic kidney disease, who have higher plasma homocysteine levels than the general population, have also not shown a benefit of B vitamins in reducing cardiovascular morbidity and mortality [71,72]. Despite a significant lowering of plasma homocysteine level by >25% from baseline values, homocysteine lowering did not affect outcomes. A recent meta-analysis of 12 randomized controlled trials that included over 47,000 subjects also concluded that homocysteine lowering did not decrease the occurrence of myocardial infarction, stroke, or all-cause mortality [2].

Overall, the numerous clinical trials suggest a relation of plasma homocysteine level to cardiovascular outcomes; however, there is no evidence of any benefit in the use of folate or other B vitamins on cardiovascular outcomes.

## 7. Methionine-Homocysteine Cycle, Folate Metabolism, and Heart Failure: Novel Cardiovascular Targets for Nutritional Manipulation

### 7.1. Clinical Heart Failure and B-Vitamin Deficiency

Heart failure is a highly prevalent condition resulting from multiple cardiac pathologies, and is associated with significant morbidity and mortality. Approximately 5.7 million individuals in the U.S. have heart failure, and approximately 50% of people with heart failure die within five years of initial diagnosis [73]. Several features of heart failure increase likelihood of nutrient deficiencies, including that of B vitamins [13]. Heart failure is more common in older individuals, and in advanced stages may be associated with cachexia. Hospitalized patients with heart failure are reported to have lower levels of B vitamins [74]. Small clinical studies in hypertension and heart failure have suggested that diuretic use is associated with lower B vitamin levels and higher plasma homocysteine levels [75,76]. Brady and coworkers, in their study of 10 subjects with heart failure and 2 controls, demonstrated that serum folate levels were significantly lower in heart failure patients [77]. Studies that measured daily intake of multiple micronutrients in older subjects have shown a decreased intake of folate in both older heart failure patients as well as in older control subjects [78,79]. A recent study by Catapano and colleagues demonstrated that folate intake was significantly reduced in heart failure subjects compared to controls [80], suggesting that in addition to the lower intake seen in the older age group commonly affected by heart failure, micronutrient deficiencies may be associated with the heart failure syndrome itself.

### 7.2. Clinical and Epidemiologic Evidence Linking Hyperhomocysteinemia to Heart Failure

Multiple small clinical studies have demonstrated that blood homocysteine levels are elevated in patients with heart failure compared to control population [81,82,83,84]. Blood homocysteine level is also related to the clinical severity of heart failure [85]. Small, prospective, clinical studies have also demonstrated that in patients with heart failure, the blood homocysteine level is predictive of adverse outcomes [86,87]. Since heart failure is associated with structural and functional changes in the heart, multiple studies have examined the relationship between blood homocysteine level and cardiac structure and function. Several small clinical studies have shown an association of plasma homocysteine level with left ventricular ejection fraction [85,86,88], and with increased left ventricular mass and volumes [89]. Nasir and colleagues utilized magnetic resonance imaging to examine the correlation of regional left ventricular function to blood homocysteine level in 1178 participants of the Multi-Ethnic Study of Atherosclerosis [90]. An elevated plasma homocysteine level was found to be associated with reduced regional left ventricular function in this analysis. Sundstrom and colleagues analyzed the relation of plasma homocysteine level to echocardiographic parameters in 2697 Framingham Heart Study participants without heart failure [91]. Homocysteine level was significantly associated with left ventricular mass and wall thickness only in women in this relatively healthy cohort. Another report from the Framingham Heart Study examined the relation of plasma homocysteine level to the incidence of heart failure over an eight-year follow-up period in 2491 adults with no evidence of heart failure or myocardial infarction [92]. Plasma homocysteine levels greater than the sex-specific median value were associated with a significantly increased risk of heart failure in both men and women. Cumulatively, these studies suggest a strong association of an elevated plasma homocysteine level with the risk of developing heart failure in the general population, as well as with worsened outcomes in patients with heart failure.

### 7.3. Preclinical Evidence Linking the Methionine-Homocysteine Cycle and Heart Failure

Studies conducted in our laboratories and those of others have shown that perturbation of the methionine-homocysteine cycle leads to adverse effects on myocardial structure and function. A report from our laboratory demonstrated that short-term (10 weeks) hyperhomocysteinemia induced by dietary supplementation of homocystine led to worsened myocardial fibrosis and diastolic dysfunction in spontaneously hypertensive rats [93]. We have shown that myocardial fibrosis develops in response to hyperhomocysteinemia in the absence of hypertension [94]. These changes were not the result of hypertension, since the diet did not change blood pressure during six months of treatment [95]. Longer term treatment (20 weeks) led to further myocardial fibrosis and accelerated progression to systolic dysfunction in spontaneously hypertensive rats [96]. Interestingly, these effects were reversible by anti-oxidant therapy (vitamins C and E) in the presence of continued diet-induced elevation of homocysteine levels [28]. Tyagi and colleagues have also demonstrated an effect of hyperhomocysteinemia on myocardial matrix and function [97]. Similar adverse effects on cardiac structure have been reported by Walker and colleagues [98]. In keeping with the results in rodent models suggesting an association between hyperhomocysteinemia and myocardial matrix deposition, a recent report from the Framingham Heart Study showed that plasma homocysteine level was significantly associated with plasma markers of collagen metabolism [99].

### 7.4. Effect of Homocysteine-Lowering Therapy on Myocardial Structure and Heart Failure

Clinical trials of homocysteine-lowering therapy have focused on atherothrombotic events and not heart failure, as described above. Herrmann and colleagues examined the effect of folate supplementation on *N*-terminal pro-brain natriuretic peptide (NT proBNP)—a marker of heart failure—in healthy subjects [100]. The authors treated 61 healthy subjects with 0.4, 1.0 or 5.0 mg/day folaic acid for two months. Although NTproBNP levels did not decrease in the entire group in response to any dose of folate, subjects with higher NT proBNP (>40 ng/L) at baseline demonstrated a 20% decrease in levels concomitant with a 15% decrease in homocysteine levels after two months of therapy. Witte and colleagues conducted a clinical study in which 30 patients with heart failure were randomized to receive a combination of vitamin B_6_, folate, and vitamin B_12_, along with calcium, magnesium, zinc, copper, selenium, vitamin A, thiamine, riboflavin, vitamin C, vitamin E, vitamin D, and Coenzyme Q_10_, or placebo [101]. After nine months of treatment, high-dose micronutrient supplementation was shown to improve left ventricular volumes and ejection fraction, as well as quality of life. It is not clear from this small study as to which component of the treatment was effective, or what the biologic mechanism of benefit was. We recently reported a substudy of the above described trial of homocysteine-lowering in subjects with advanced kidney disease and elevated homocysteine levels [6]. In this study, we analyzed the effects of homocysteine-lowering with high dose B-vitamin therapy on clinical outcomes and parameters of cardiac structure and function measured by echocardiography. B-vitamin treatment did not affect heart failure outcomes. Interestingly, left atrial size, a surrogate marker of cardiac filling pressures and function, was adversely affected (increased) by high dose treatment with folate, vitamin B_6_, and vitamin B_12_. These studies suggest that there may be a relation of a perturbed methionine-homocysteine cycle to myocardial function, and that further research would be important in elucidating the relation of B vitamins to heart failure outcomes.

## 8. Methoxistasis—A Novel Paradigm

There is strong evidence of a correlation between an elevated plasma homocysteine level and both atherothrombotic cardiovascular disease and heart failure. However, clinical trials that utilized B vitamins singly or in combination, and in low or high doses, have not shown any benefit in lowering atherothrombotic risk. Furthermore, studies have also shown a potential for adverse effects of B-vitamin supplementation [5,6]. The approach thus far has not taken the complex relationship between folate, one-carbon metabolism, and the methionine-homocysteine cycle described in preceding sections into consideration. We will attempt to provide a framework for further research in this area by proposing a novel paradigm, that of methoxistasis.

**Figure 3:**
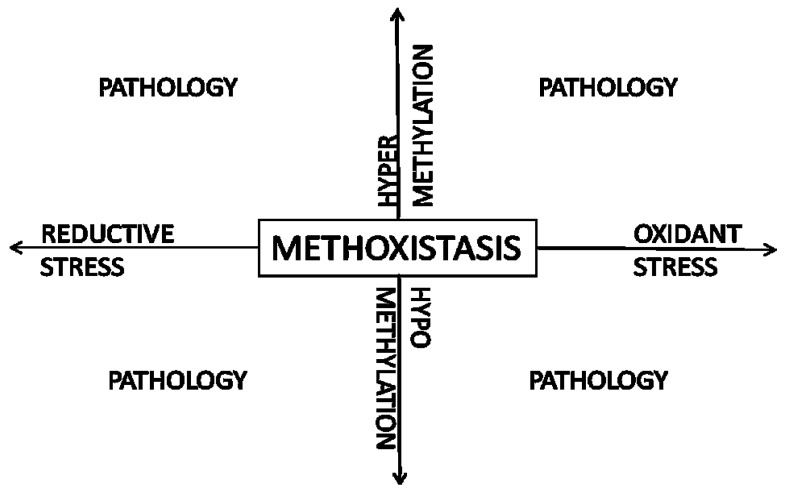
Methoxistasis: the close interrelationship between redox status and methylation balance is disturbed in pathologic states.

As shown in Figure 3, normal redox signaling and normal methylation is crucial for the maintenance of homeostasis. Excess of reactive oxygen or nitrogen species can lead to oxidant stress, a major stimulus for cardiovascular pathology [102]. Reductive stress has also been implicated in cardiovascular disease [103]. Similarly, normal methylation is crucial for normal gene expression and cellular function. Hypo- or hypermethylation can alter gene and protein function. DNA methylation, and consequent epigenetic changes, are most widely studied among methylation reactions, and are crucial determinants of embryonic development and normal somatic cellular function [104]. As detailed in the preceding sections and figures, methylation balance and redox potential are linked via the methionine-homocysteine cycle and allied folate/one-carbon metabolism. A recent study from our laboratories highlighted the close link between redox and methylation balance [105]. Selenium deficiency increased oxidant stress in the myocardium and caused myocardial fibrosis. Interestingly, modest selenium supplementation, while reducing redox levels (*i.e*., increasing/restoring reduction potential) in the myocardium, also reduced methylation potential and DNA methylation and led to the same phenotype observed in a selenium deficient state, *i.e.*, myocardial fibrosis.

Although the homocysteine level may be a general gauge of deviation from methoxistasis, it is not in itself a sufficient assessment. As shown in Figure 3, deviations from methoxistasis may take the form of oxidant stress/hypomethylation, oxidant stress/hypermethylation, reductive stress/hypomethylation, and reductive stress/hypermethylation. This may depend on genetic and environmental factors such as the MTHFR polymorphism, age, sex, intake of alcohol, malnutrition, sulfur-containing aminoacid intake, *etc.* Hence, an abnormal plasma homocysteine level only uncovers an underlying disturbance of methoxistasis that needs to be further characterized. It follows that lowering the plasma homocysteine level utilizing folate and other B vitamins without understanding the disturbance in methoxistasis may not be effective and may indeed be harmful as shown by clinical trials. For example, folate supplementation could increase methionine and SAM levels and, thereby, methylation, while the combination of folate, vitamin B_12_, and vitamin B_6_ could promote both increased methylation as well as increased transsulfuration and possible reductive stress. Hence, the approach to an elevated plasma homocysteine level may be determined by analyzing the underlying abnormalities in redox status and methylation and utilizing B vitamins, anti-oxidants, or micronutrients, such as selenium, singly or in combination to restore methoxistasis.

## 9. Conclusions

The data presented above, while clearly demonstrating a relationship of an elevated plasma homocysteine level to cardiovascular disease, fail to show any benefit of unselected folate and B-vitamin supplementation. Rather than conclude that the association of hyperhomocysteinemia is not causal, we would like to propose that the plasma homocysteine level is a marker of perturbation of methoxistasis. Instead of focusing on lowering the homocysteine level, further pre-clinical and clinical research should concentrate on understanding the disturbance of redox-methylation balance seen in hyperhomocysteinemic states and on approaches to restoring methoxistasis. Based on such investigations, clinical strategies to mitigate cardiovascular risk could be developed. In addition to atherothrombotic cardiovascular disease, heart failure is also an attractive target for nutritional strategies to restore methoxistasis. With the rapid advances in -omics technologies, restoring methoxistasis by personalized medicine strategies may be an option in the future of cardiovascular prevention and therapeutics.
